# Advancing mental health for all: WPA 2023–2026 action plan on clinical education and healthy lifestyles

**DOI:** 10.1007/s40211-025-00545-3

**Published:** 2025-08-26

**Authors:** Danuta Wasserman

**Affiliations:** 1 National Centre for Suicide Research and Prevention of Mental Ill-Health (NASP), Department of Learning, Informatics, Management and Ethics, Karolinska Institutet, Stockholm, Sweden; 2 World Psychiatric Association (WPA), Geneva, Switzerland

**Keywords:** WPA strategy 2023–2026, Mental health promotion, Sustainable development goals, Suicide prevention, EDIT principles, WPA-Aktionsplan 2023–2026, Förderung psychischer Gesundheit, Nachhaltige Entwicklungsziele, Suizidprävention, Gesunder Lebensstil, EDIT-Prinzip

## Abstract

The World Psychiatric Association (WPA) is dedicated to advancing mental health through evidence-based and inclusive initiatives. Its 2023–2026 Action Plan emphasizes prevention, early intervention, and comprehensive care, aligned with the United Nations 17 Sustainable Development Goals to address broader social determinants of mental health. An important focus of the Action Plan is promoting healthy lifestyles as a fundamental component of mental well-being. Through the Healthy Lifestyle Hub, the WPA promotes the benefits of physical activity, good nutrition, and sleep hygiene in preventing and managing mental health conditions. Complementing these efforts, the Specialist Corner, through dissemination of scientific advances and their clinical applications, facilitates knowledge exchange among clinicians, researchers, and policymakers, while the WHO Brief Motivational Intervention and Contact program (BIC), comprising long-erm regular follow-up of suicide attempters after hospital discharge, enhances suicide prevention. Guided by the EDIT principle—Equality across genders, ages, and ethnicities; Developmental stages from childhood to adulthood and beyond; Inclusion; and Transcultural awareness—the WPA ensures that mental health strategies are inclusive, equitable, and culturally responsive. By integrating scientific advancements with lifestyle-based interventions, the WPA Action Plan serves as a framework for global psychiatry, advocating sustainable and comprehensive approaches to mental well-being.

## Abstract (de)

Die World Psychiatric Association (WPA) setzt sich für evidenzbasierte und inklusive Initiativen ein, um psychische Gesundheit zu fördern. Der Aktionsplan der Jahre 2023–2026 betont Prävention, frühzeitige Interventionen und umfassende Versorgung, in Abstimmung mit den 17 Zielen für nachhaltige Entwicklung (Sustainable Development Goals) der Vereinten Nationen, um die breiteren sozialen Aspekte von psychischer Gesundheit zu berücksichtigen. Ein wichtiger Schwerpunkt des Aktionsplans ist die Förderung eines gesunden Lebensstils als zentrale Komponente psychischen Wohlbefindens. Durch den Schwerpunkt auf gesunden Lebensstil fördert die WPA die Vorteile von körperlicher Aktivität, gesunder Ernährung und Schlafhygiene, um psychischen Erkrankungen vorzubeugen. Eine Plattform namens „Specialist Corner“ ergänzt diese Bemühungen durch die Verbreitung wissenschaftlicher Erkenntnisse und deren klinischer Anwendung, durch Wissensaustausch zwischen Klinikern, Forschern und politisch Verantwortlichen. Das wird ergänzt durch das Brief Motivational Intervention and Contact Programm (BIC) der Weltgesundheitsorganisation (WHO), das durch eine langdauernde Begleitung von Personen nach der Krankenhausentlassung wegen eines Suizidversuchs weiteren Suizidversuchen vorbeugen will. Die WPA arbeitet nach dem EDIT-Prinzip (*Gleichheit* zwischen Geschlechtern, Altersgruppen und ethnischen Gruppen; Berücksichtigung der *Entwicklung* über die Lebenszeit; *Inklusion*; Bewusstsein für *transkulturelle* Aspekte) um sicherzustellen, dass alle Strategien inklusiv, gerecht und kulturell passend sind. Durch die Kombination von wissenschaftlichem Fortschritt mit Lebensstilinterventionen bietet der WPA-Aktionsplan einen Rahmen für die weltweite Psychiatrie, der nachhaltige und umfassende Ansätze für psychisches Wohlbefinden fördert.

## Introduction

The World Psychiatric Association (WPA) recognizes that the well-being of individuals hinges on evidence-based and inclusive mental health promoting initiatives. Central to WPA’s commitment to mental health is its multifaceted Action Plan for 2023–2026, which underscores the importance of prevention, early intervention, and holistic care for those experiencing mental health problems [1]. These overarching goals align with the broader global agenda of improving health across the lifespan, ensuring equity, and fostering a sustainable environment.

### United Nations Sustainable Development Goals

Recognizing that mental health is woven into a wide range of social, economic, and environmental determinants, the WPA’s Action Plan 2023–2026 closely aligns with the 17 United Nations Sustainable Development Goals (SDGs) [2]. The SDGs, ranging from eradicating poverty (Goal 1) and hunger (Goal 2), to promoting quality education (Goal 4) and ensuring sustainable cities and communities (Goal 11), reflect a global commitment to fostering conditions in which all individuals can thrive [2]. Mental health is integral to each of these goals, as it influences and is influenced by factors such as education, employment, housing, and social support [3]. For example, quality education (Goal 4) provides children and adolescents with the foundational skills and emotional resilience required to navigate challenges in adulthood. Likewise, decent work and economic growth (Goal 8) are crucial for mental well-being because stable employment can reduce stress, improve self-esteem, and facilitate social integration.

The promotion of good health and well-being (Goal 3) explicitly acknowledges mental healthcare access, parity, and the integration of services across primary and secondary healthcare settings. Climate change (Goal 13) is a pressing global issue that powerfully intersects with mental health [4]. The psychosocial effects of climate change can be profound; climate-related stressors, such as extreme weather events, forced migration, and resource scarcity, increase psychological distress, increase rates of depression and anxiety, and challenge existing mental health infrastructure, particularly for marginalized communities that lack adaptive resources to cope with increased environmental volatility [4]. These challenges are often compounded by wars, armed conflicts, and terrorist activities, which further displace populations, destroy healthcare infrastructure, and heighten trauma exposure across all age groups. In response to these converging global threats, the WPA’s Action Committee on Responses to Emergencies (ACRE) plays a crucial role in coordinating rapid mental health responses, supporting professionals in crisis settings, and advocating for integrated emergency preparedness strategies that prioritize mental health.

In alignment with the SDGs, the WPA Action Plan 2023–2026 reinforces a shared vision of sustainable development that prioritizes human physical and mental well-being equally [1, 5, 6]. Furthermore, the WPA Action Plan underscores the need for proactive strategies designed to fill gaps in mental health services and amplify existing strengths in psychiatric research and practice, addressing the fact that nearly 13% of the global population is affected by mental health problems, yet only 2% of health expenditure is devoted to this challenge [7]. In addition, the WPA aims to translate the latest research into clinical practice by means of the Specialist Corner, enhance prevention efforts through its Healthy Lifestyles Hub, and advance suicide prevention with the World Health Organization’s (WHO) Brief Motivational Intervention and Long-Term Regular Follow-Up Contact Program (BIC; [1, 7]).

### Specialist Corner: advances of science and their application in clinical practice

The Specialist Corner: Advances of science and their application in clinical practice is an innovative platform where leading clinicians and researchers share expertise in novel therapies, technological developments, and best practices [1]. This corner serves as a link for ongoing professional education.

The WPA Specialist Corner’s activities focus on the following diagnostic categories: schizophrenia, other psychoses, affective disorders, intellectual impairment, dementia, substance use/abuse, and anxiety disorders presented in the webinar form by recognized authorities in science and clinical work and commented by leaders of expert groups in key cross-cutting areas, such as comorbidity, public mental health, partnership with persons who have lived experience, ethics and legal issues, digitalization, physical exercise, lifestyle, and culture in order to guide WPA membership in the best way forward in treatment, prevention, and rehabilitation [1]. Together, they facilitate the application of scientific advancements in psychiatric treatment, ensuring that guidelines and interventions are shaped by evidence-based and diverse perspectives and are tailored to the varying needs of patients.

### Brief Motivational Intervention and Long-Term Regular Follow-up Contact Program (BIC)

The WHO BIC program, spearheaded by the WPA, is a suicide prevention initiative that integrates clinical care with broader public mental health approaches, harnessing both professional healthcare systems and community volunteers to provide support [7]. The WHO BIC program offers a pivotal suicide prevention strategy to follow, in a structural way for a longer period, individuals who present to emergency departments after a suicide attempt [7]. The WHO BIC program is a practical resource-sensitive intervention that can be implemented in diverse healthcare settings.

Further information on the WHO BIC program—including implementation guidelines, clinical protocols, training videos, links to pre–post evaluation studies and randomized controlled trials (RCTs)—is available on the WPA website as part of the resources supporting the WPA Action Plan 2023–2026.

### Promotion of healthy lifestyles

Alongside the aforementioned initiatives, the WPA 2023–2026 Action Plan includes the promotion of healthy lifestyles as a crucial adjunct to existing treatments, including pharmacological, psychosocial, and psychotherapeutic interventions, to improve mental health [1, 5]. A growing body of evidence indicates that lifestyle factors such as physical activity, good nutritional habits, and sleep hygiene play a substantive role in the management and prevention of mental health problems [8–14]. Studies have shown that patients who adopt healthier lifestyle habits often experience reduced symptom severity, improved treatment adherence, and an overall better quality of life [8–14].

#### Physical activity

Physical activity is among the most well-documented protective factors for mental health, with an array of studies indicating that regular exercise can alleviate depressive symptoms, reduce stress, and enhance cognitive function [8–11]. Physical activity enhances mental health through interconnected biological and psychosocial mechanisms, including optimized neuroendocrine responses to stress, reduced systemic inflammation, and increased neural plasticity [8]. These processes collectively contribute to resilience against psychiatric symptoms across the lifespan, reinforcing the role of physical fitness as a protective factor against mental health disorders. Mental health problems often begin early in life, with 50% emerging by age 14 and 75% by age 24, and 70% of preventable adult deaths from noncommunicable diseases are linked to risks and unhealthy behaviors developed during adolescence [9]. Regular physical activity plays a crucial role in improving adolescent mental health [10]. Encouraging movement and limiting sedentary time can serve as an effective strategy for promoting well-being and preventing mental health challenges in young people [10]. WPA’s Healthy Lifestyle Hub, highlighted in the Action Plan, provides guidance on exercise regimens tailored to specific populations, including older adults, adolescents, and individuals with comorbid chronic diseases [1, 5]. These resources empower psychiatric professionals to advise patients on evidence-based exercise interventions, ensuring that each individual’s unique circumstances and physical capacity are duly considered [1, 5]. Beyond clinical benefits, interventions that foster community-based physical activities also reduce social isolation, a key driver of poor mental health outcomes [1, 5]. These WPA-led efforts converged with the broader aim of strengthening social support networks and promoting a culture of shared responsibility for mental wellness.

Further resources—such as instructional videos produced by Karolinska Institutet (Stockholm, Sweden), which guide psychiatric staff and patients through daily physical activity routines—are available on the WPA Healthy Lifestyles Hub. These tools, featured as part of the WPA Action Plan 2023–2026, offer practical and evidence-informed guidance on incorporating physical activity into mental healthcare in clinical practice.

#### Nutrition

Similarly, nutrition plays a crucial role in mental healthcare, with evidence supporting the idea that nutrition influences mood, cognitive function, and stress reactivity [12, 13]. Specific research has attributed the protective benefits of diets rich in fruits, vegetables, whole grains, and lean proteins to the anti-inflammatory and neuroprotective properties of key nutrients [13]. There are still gaps in understanding the precise mechanisms linking diet and mental health, particularly in cognitive aging and neurodegenerative diseases. However, research shows that anti-inflammatory dietary patterns, such as the Mediterranean diet (MD), low-salt diet, and the Dietary Approaches to Stop Hypertension (DASH), may play a neuroprotective role [13]. These diets are rich in omega‑3 fatty acids, antioxidants, and polyphenols, which help reduce neuroinflammation—a key factor in cognitive decline and Alzheimer’s disease [13]. Additionally, emerging evidence suggests that diet influences cognitive health through indirect immune pathways, including interactions with the gut microbiome and systemic inflammation [13]. While more human studies and neuroimaging research are needed to fully understand these links, current findings support the role of diet in promoting brain health and mitigating cognitive decline.

By collaborating with dietitians and public health experts, the WPA’s Healthy Lifestyle Hub generates practical tools and workshop materials that align dietary recommendations with local food cultures [5]. Such culturally sensitive dietary interventions can be a critical part of integrated mental healthcare models, offering patients tangible and sustainable self-management strategies. Nutritional videos have been produced at the WPA in collaboration with the University of Campania “Luigi Vanvitelli” to highlight the role of diet in mental well-being. These videos showcase specific foods and dietary patterns that support brain function, demonstrating meal preparation, balanced nutrition strategies, and the benefits of key nutrients for mental well-being. Designed for young people and healthcare professionals, they serve as an educational tool to promote awareness about healthy eating habits as part of a comprehensive mental health strategy.

The nutrition-focused videos produced by the University of Campania Luigi Vanvitelli—including educational dialogues and practical advice tailored for adolescents, young adults, and clinical settings—are available on the WPA Healthy Lifestyles Hub.

#### Sleep

Sleep hygiene, another pillar of WPA’s healthy lifestyle strategy, has been repeatedly associated with mental health outcomes. Poor sleep is strongly correlated with an increased risk of depression, anxiety, and psychosomatic complaints [14]. Building awareness among mental health professionals regarding the role of circadian rhythms, sleep architecture, and environmental factors (e.g., light exposure and screen time) is essential. The WPA, through its Action Plan and Healthy Lifestyle Hub, focuses on producing evidence-based recommendations that clinicians can tailor to individual patients’ needs [5]. Good sleep practices, such as regular sleep scheduling, limiting caffeine and screen use before bedtime, and ensuring a comfortable sleep environment, can significantly bolster the effects of pharmacological and psychotherapeutic interventions [14]. Moreover, when effectively implemented, these simple yet impactful changes mitigate the risk of relapse and cultivate a foundation for long-term mental well-being.

### Healthy lifestyles for psychiatrists

The WPA Action Plan for 2023–2026 also emphasizes the promotion of healthy lifestyles to enhance mental health among psychiatric professionals. This initiative acknowledges that psychiatrists often face demanding workloads, making it challenging to prioritize their own well-being. By integrating healthy lifestyle practices, such as regular physical activity, psychiatrists can improve their own mental health, serve as role models for patients, and effectively incorporate lifestyle interventions into clinical practice. This holistic approach aims to foster a culture of health within psychiatric care, benefiting both healthcare providers and those they serve.

Incorporating physical activity as a shared experience between psychiatric staff and patients offers additional benefits beyond improved physical and mental health. Engaging in exercise together can strengthen the connection between healthcare providers and patients by fostering a sense of unity, improving communication, and increasing empathy. This shared activity helps reduce hierarchical barriers, creating a more personalized and supportive therapeutic environment. At the same time, it enhances the well-being of participating staff, reinforcing the importance of a healthy lifestyle within psychiatric care settings.

By advocating for healthy lifestyle changes, the WPA advocates for the reinforcement of treatments with healthy lifestyle habits [6]. This emphasis ensures that patients receive a comprehensive care strategy—one that mitigates risk factors, nurtures resilience, and recognizes the interplay of the biological, psychological, and social dimensions of mental health.

### EDIT principle

Underpinning all current initiatives under the WPA’s 2023–2026 Action Plan, including its alignment with the UN Sustainable Development Goals, is the EDIT principle [15]. EDIT stands for (a) *e*quality across genders, ages, and ethnicities on the basis of equality in possibilities for education, work opportunity, housing, access to mental health services etc., (b) observing that mental health needs vary across the lifespan and *d*evelopmental stages from childhood to adulthood and beyond; (c) *i*nclusion of under-represented groups; and (d) *t*ranscultural awareness and perspectives [15]. Introducing the EDIT principles in the WPA action plan ensures increased awareness that research and clinical practice are interlinked with social, cultural, economic, and gender-based determinants of mental health, helping to identify and address critical gaps in knowledge and practice.

Scientific excellence is always the cornerstone of all WPA research and clinical efforts. In the choice of the content of the scientific program of World Congresses, the scientific merit of the submitted proposals also has an overriding priority, coupled with the consideration of geographical spread and over- and underrepresented topics. This approach aims to enhance the visibility of diverse voices and gaps in psychiatric research. By embracing both scientific excellence and the EDIT framework, the WPA positions itself at the forefront of global mental health leadership by increasing visibility and raising awareness of current gaps in knowledge and how they influence clinical practices.

## Conclusion

The WPA Action Plan 2023–2026 serves as a dynamic roadmap, evolving in response to emerging scientific evidence, stakeholder input, and changing global realities. Its success hinges on strong partnerships between clinicians, researchers, patients, policymakers, and community members. As part of this effort, the WPA remains resolute in its commitment to scientific integrity by using evidence-based methods, ethical conduct, and respect for human rights—values that resonate in every dimension of the organization’s work. Through consistent advocacy, groundbreaking research, and collaborative engagement, WPA stands poised to guide global psychiatry into an era defined by innovation, inclusivity, and holistic physical and mental well-being.
